# Depolymerized Chitosan-g-[Poly(MMA-co-HEMA-cl-EGDMA)] Based Nanogels for Controlled Local Release of Bupivacaine

**DOI:** 10.3390/ijms242216470

**Published:** 2023-11-17

**Authors:** Sivagangi Reddy Nagella, Soojeong Choi, Soo-Yong Park, Chang-Sik Ha, Youngmi Jung, Ramesh Kumar Chitumalla, Joonkyung Jang, Ji-Young Yoon, Ildoo Chung

**Affiliations:** 1 Department of Polymer Science and Engineering, Pusan National University, Busan 46241, Republic of Korea; sivagrnagella@gmail.com (S.R.N.); csha@pusan.ac.kr (C.-S.H.); 2 Department of Biological Sciences, College of Natural Science, Pusan National University, Busan 46241, Republic of Korea; y.jung@pusan.ac.kr; 3 Department of Nanoenergy Engineering, Pusan National University, Busan 46241, Republic of Korea; rameshchitumalla@gmail.com; 4 Department of Dental Anesthesia and Pain Medicine, School of Dentistry, Pusan National University, Gyeongsangnam-do, Yangsan 50612, Republic of Korea; 5 Dental Research Institute, Pusan National University Dental Hospital, Gyeongsangnam-do, Yangsan 50612, Republic of Korea

**Keywords:** chitosan, nanogel, bupivacaine, pain management, local drug delivery

## Abstract

This study is designed to formulate and characterize chitosan-based nanogels that provide the controlled delivery of anesthetic drugs, such as bupivacaine (BPV), for effective postoperative pain management over prolonged periods of time. Drug carriers of chitosan/poly (MMA-co-HEMA-cl-EGDMA) (CsPMH) nanogels were prepared by varying the composition of comonomers such as MMA, HEMA, and redox initiator CAN. The nanogels were then characterized using FTIR, TGA, SEM, and TEM. The CsPMH nanogels showed greater encapsulation efficiencies from 43.20–91.77%. Computational studies were also conducted to evaluate the interaction between the drug and CsPMH nanoparticles. Finally, BPV-loaded nanoparticles were used to examine their in vitro release behavior. At pH 7.4, all the drug carriers displayed the “*n*” value around 0.7, thus the BPV release follows anomalous diffusion. Drug carrier 7 demonstrated a steady and sustained release of BPV for approximately 24 h and released about 91% of BPV, following the K-P mechanism of drug release. On the other hand, drug carrier 6 exhibited controlled release for approximately 12 h and released only 62% of BPV.

## 1. Introduction

Postoperative pain management is an inseparable part of the recovery process and is very essential to enhance the outcomes of the surgery in terms of maximizing recovery and reducing the duration of hospitalization [1,2]. However, pain relief by systemic administration of analgesic drugs may lead to severe adverse effects on the central nervous system, gastrointestinal system, and other organs [3,4]. Thus, a safe medical procedure called interventional pain management is required to relieve the pain, in which, one or more pain management techniques such as tissue infiltration, nerve blocks, and neuraxial anesthesia are used with the aid of local anesthetic drugs [5]. Bupivacaine (BPV) is a commonly used long-acting local anesthetic drug used for perioperative pain management, which arrests nerve signal conduction by inhibiting sodium influx, which is essential for the generation and propagation of action potentials in nerve cells [6]. Further it provides extended-duration anesthesia for local infiltration, a peripheral nerve block. Although, BPV is more soluble in organic solvents like dimethyl sulfoxide, dimethyl formamide, and ethanol, it is moderately soluble in water due to its polarity and suitable pKa (7.8), which is close to the physiological pH of 7.4 [7]. However, BPV.HCl is highly soluble in water. Thus, amphiphilic polymeric systems are required for optimum loading and efficient drug release [8].

Nowadays a combination of local anesthetic drugs is used increasingly in epidural and spinal anesthesia. To reduce the onset time and enhance the quality of epidural anesthesia, BPV is formulated with epinephrine and sodium bicarbonate. [9]. However, the making of formulations of mixed drugs sometimes raises chemical stability issues [10]. The chemical stability is one of the key factors for assessment of pharmaceutical properties, activity, and selectivity of drug formulations. BPV is chemically stable up to one month at room temperature and 24 h at 45 °C and its alkaline (sodium bicarbonate) solutions also stable up to 6 h. The bupivacaine formulations are usually administered via intravenous (IV), intra-arterial (IA), epidural, and intrathecal routes in dogs [11]. However, the intravenous (IV and IA routes) administration usually has a high risk of cardiovascular problems in humans and animals [12]. Thus, it is injected into humans using the epidural, spinal, intradermal, or subcutaneous routes, whereas the epidural delivery of BPV is very efficient and does not cause many adverse effects. Sometimes BPV can be continuously instilled into the surgical wounds of cesarean delivery to reduce the inflammation or tissue damaging effects by anesthetic-induced tissue toxicity [13]. Thus, the main challenge of BPV release is the reduction of potential toxic and adverse effects by improving the effective passage through the blood–brain barrier. This can be significantly overcome by using nano drug carriers via controlled and efficient release of drugs [14]. However sometimes non degradable carrier polymer microparticles cause myotoxicity compared to 0.5% BPV.HCl [15], thus the selection of polymers becomes very important in the anesthetic drug release. Due to these difficulties finding the ideal delivery system having long-acting delivery with low side effects for perioperative pain treatment is challenging.

Therefore, efficient drug delivery systems (DDS) should be evolved to increase the bioavailability of drug molecules at target tissues. Because drug delivery efficiency is an important parameter in the treatment of disease [16], the development of a highly efficient DDS is a challenging task, as most pharmaceuticals face lower adsorption due to large molecular weight and slow onset of action due to systemic drug admission, while non-selectivity of drug delivery causes potential side effects in the off-target tissues [17].

Bioavailability is not only an important gauge in the adsorption of drug moiety, which measures both extent and rate of accessing the site of action to affect the target tissue, but also an essential tool in pharmacokinetics to measure dosage forms [18]. Poor in vivo activity of the active drug is mainly attributed to its low bioavailability [19], which usually depends on the route of administration and physiology and the metabolism of the organ [20]. Furthermore, the systemic administration of drugs always suffers from their low bioavailability, which leads to repeated dosage [19], whereas controlled delivery systems offer an alternative approach, that regulates the bioavailability of therapeutic agents in an optimal range in a predefined manner. However, localized drug delivery which is referred to as the release and absorption of active drug molecules or their subsequent transport through biological membranes to the site of action [16,21], often provides improved bioavailability of the drug moiety at the site of action with minimized systemic side effects [22]. Even though systemic delivery is prominent for most diseases, localized controlled release systems provide an improved outcome for certain treatments like discrete tumors, local anesthesia, and deep-seated infections [23].

Nano drug delivery systems are promising candidates for sustained and controlled delivery of therapeutics. Recently, Isabel et al. developed cleavable and thermo-responsive CuS-poly(ethylene glycol) hybrid nanoparticles for on demand release of BPV by applied NIR light stimulation [24]. Nadri et al. achieved magnetic aided release of BPV in rats by using magnetic polymer nanogels formed by NIPA–methacrylic acid–bupivacaine complexes [25]. Similarly, Todd et al. developed magnetically triggered nanocomposite membranes with ethyl cellulose and N-isopropyl acrylamide-co-N-isopropyl methacrylamide-co-acrylamide nanogels [26]. Park et al. developed biodegradable polyfumarateurethane nanoparticles and used them for release of BPV; they achieved sustained release up to 7 h [7]. The evaluation of nano drug delivery systems and the knowledge of the precise dosage of the medication produced by those systems are also crucial. However, drug delivery by nanoparticles suffers from the lack of a standard method for in vitro release evaluation. Assaying the drug released in in vitro studies suffers mainly from numerous practical challenges [27]. Three different in vitro acellular procedures are currently available: dialysis membrane, continuous flow, and sample and separate [28]. For fast and complete dissolution of drug dosage forms, sink conditions are very important, however, maintenance of non-sink conditions leads to slow dissolution rates and discriminatory dissolution profiles for poorly soluble drugs due to near saturation solubility. Every method has its own pros and cons, but a widely useful method is sample and separate due to its direct approach to monitor in vitro release [28]. It has some practical challenges such as interference of drug release during the separation of nano particles, destabilization of nanoparticles during the long and high-speed centrifugation process, and a clogging effect of filters due to the adsorption of the drug on to the filters during the separation process [29].

Biopolymer-based microgels/nanogels have recently demonstrated a great opportunity in drug and gene delivery applications [30]. Biopolymers possess a high content of functional groups including hydroxyl, amino, and carboxylic acid groups, which can be utilized in the making of drug and bioconjugates to target tissues and for cross-linking of polymer networks. Additionally, they have suitable physicochemical properties along with biocompatibility and biodegradability [31]. Recently, Park et al. developed gene carrier nanoparticles based on L-tyrosine polyurethane, which showed a higher transfection efficiency of genes [32]. Similarly, carbopol-g-methacrylic acid pH responsive nanogels were developed for gastroprotective delivery of ketoprofen and they achieved first order release for about 5 h [33]. Dual stimuli responsive hollow nanogel spheres were developed with acrylic acid and N-isopropyl acrylamide (NIPA) [34]. These hollow nanogels displayed rapid release of DOX at pH 5.0, due to extensive disruption of acrylic acid–DOX complexes. Water soluble pH responsive chitosan amphiphilic core-shell nanoparticles were developed using chitosan and polyethylene glycol methacrylate for water insoluble anticancer paclitaxel and antifungal berberine drugs [35]. These nanoparticles maintained a steady state of drug release of greater than 10 days and S. ampelinum inhibition capacity for about 30 days.

Chitosan (Cs), one of the most abundant naturally occurring cellulose polymers, which is generally obtained by the deacetylation of chitin extracted from the shells of crustaceans [36,37], has been used as an effective material in various applications including DDS. It is an amino polysaccharide made up of the copolymers of D-glucosamine and N-acetylated D-glucosamine which are linked via β-1,4-glycosidic linkages [37], and used because of the many attractive properties such as excellent biocompatibility, biodegradability, muco-adhesivity, non-toxicity, and non-immunogenicity. Furthermore, Cs could be depolymerized by treating with NaNO_2_ to obtain highly soluble lower molecular weight polymer chains without any structural changes [38]. The chitosan oligomers are derived from the depolymerization of medium and low molecular weight chitosan. These are attractive for gene and drug delivery applications due to their water solubility and ease of altering properties for functionalized materials [39]. Therefore, we prepared the amino functionalized and MMA-co-HEMA grafted CsPMH nanogels using EGDMA and CAN. Further the nanogels fabricated using the preferred acrylic monomers such as methyl methacrylate (MMA) and 2-hydroxyethyl methacrylate (HEMA) are biocompatible and do not display any cytotoxicity and hemolytic activity in biological systems [40]. However, the MMA monomer imparts hydrophobicity to nanogels, which is essential for large BPV loading capacities and slower-releasing properties called sustained release. Thus, the introduction of HEMA/MMA-based homo and copolymers makes the hydrogels more efficient for biomedical applications due to their biocompatibility, and large drug-loading capacity.

In this paper, we demonstrate the synthesis of CsPMH nanogels by adjusting the hydrophilic–lipophilic balance, which is essential for the sustained release of BPV, and investigate their drug delivery efficiencies under two different physiological conditions to characterize their drug release behaviors and mechanism.

## 2. Results and Discussion

### 2.1. Characterization

#### 2.1.1. FTIR

**Figure 1:**
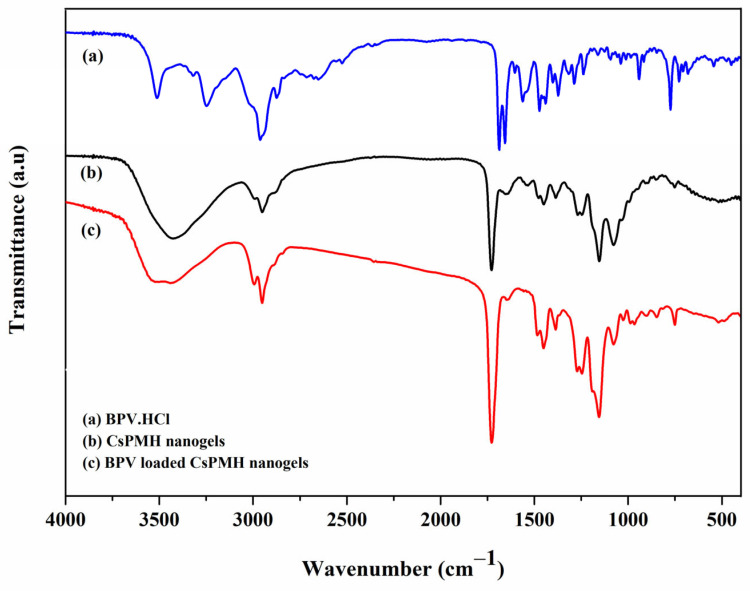
FTIR spectra of BPV.HCl, CsPMH, BPV loaded CsPMH nanogels.

The FTIR spectra of pure BPV, CsPMH nanogels and BPV loaded CsPMH were taken on the JASCO IR spectrophotometer and are presented in Figure 1. The pure BPV displays characteristic peaks related to amide N–H stretching (3321 cm^−1^, 3249 cm^−1^), aromatic C=C ring stretching (at 1685 cm^−1^), amide I and II bands (1657 cm^−1^, 1560 cm^−1^), and carbon skeletal frequencies (−2900 cm^−1^ and −1400 cm^−1^) [41].

The pure CsPMH nanogels displays predominant peaks at 3518 cm^−1^ for the –O–Hgroups, 1728 cm^−1^ for the ester C=O stretching, 1152 cm^−1^ for the C–O–H stretching vibrations of side chains of cross-linked polymer chains, 3434 cm^−1^ for N–H stretching, and merged peaks at 1728 cm^−1^ and 1640 cm^−1^ for the amide I and II of Cs. The peak at 1073 cm^−1^ corresponds to the C–O–C stretching frequency of both synthetic and natural polymer Cs. As shown in Figure 1 and Table 1, the FTIR spectrum of BPV loaded gels displays all the peaks of pure CsPMH nanogels in addition to the hump like peak at 1560–1530 cm^−1^, which belongs to amide II of BPV.

However, with stronger interactions with the loaded BPV, the amide groups of the drug-laden gel undergo a red shift, which may be explained by hydrogen bond interactions between BPV and the polymer chains. Additionally, a similar effect is observed for the –CH_2_ and –CH_3_ bending frequencies of the drug-loaded polymer network, indicating the presence of hydrophobic interactions between the polymer chains and BPV. These findings confirm the successful loading of BPV into the CsPMH nanogels.

#### 2.1.2. Thermo-Gravimetric Analysis (TGA)

**Figure 2:**
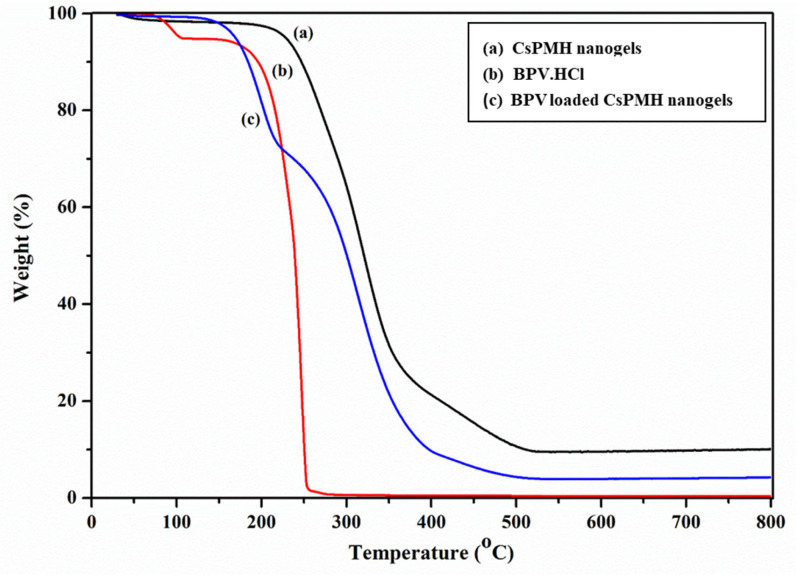
Thermograms of BPV HCl, CsPMH, BPV loaded CsPMH nanogels.

TGA is an analytical method used to evaluate thermal stability of the material by monitoring the weight change of the sample with temperature, when the sample is heated at a constant rate in a controlled environment. As shown in Figure 2 and Table 2, the three samples, pure BPV.HCl, CsPMH, and BPV loaded CsPMH, were analyzed with TGA.

The thermogram (a) of CsPMH nanogels presents three distinct weight loss steps. The first step has 1.28% of weight loss between 30–80 °C attributed to the loss of moisture from the nanogels. While the second stage was observed in the temperature range of 204–375 °C accounting for 72.44% of weight loss, which is due to the decomposition of the grafted polymer side chains and Cs [37]. The final stage of decomposition observed in the temperature range of 375–519 °C accounted for up to 15.24% of weight loss in nanogels, which is due to the decomposition of the main chain of the polymer network.

In the thermogram of BPV.HCl (b) shown in Figure 2, two stage weight loss was observed. The first stage was accounted for by the weight loss of 5.22% of water, while the second stage was accounted for by up to 93.96% of weight loss observed in the temperature range 150–283 °C, due to the thermal decomposition of BPV. However, the BPV loaded CsPMH nanogels (c), displays 27.56% of a new weight loss in the temperature range of 118–227 °C due to the loading of BPV into the nanogels, in addition to the CsPMH decomposition curves at the temperature range between 227–400 °C (61.80%) and 400–516 °C (5.80%). From the above results, it could be observed that the entrapment of BPV into the nanogels makes it less stable than pure CsPMH nanogels (a) due to its weak hydrophobic interactions between the polymer chains via intercalation of BPV in the nanogels network.

#### 2.1.3. Morphology

**Figure 3:**
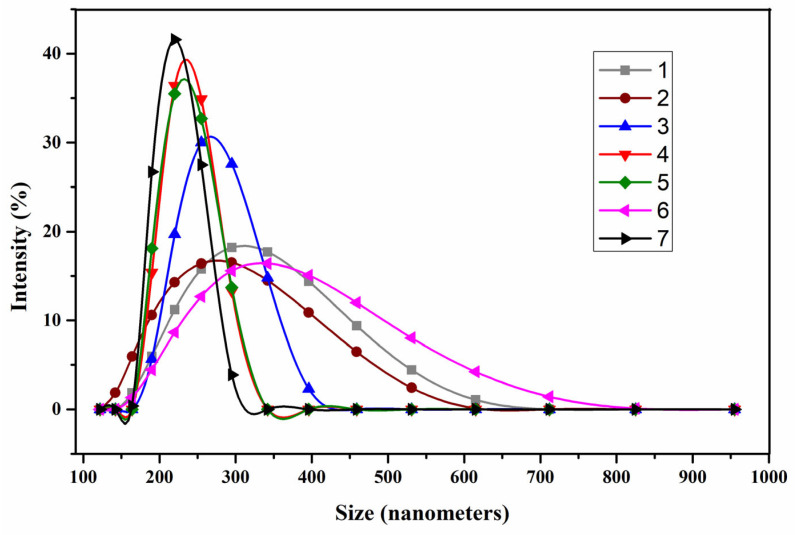
DLS histograms of different drug carriers of CsPMH nanogels.

The z-average diameters of the nanogels measured by DLS at 25 °C are presented in Figure 3, and Table 3, from which one can see that all drug carriers have a narrowly distributed diameter ranging from 130 to 310 nm, except for the drug carrier 6, which has long polymer graft chains compared to the others. Further, it can be seen that the size of the nanogels changed according to the increase of the MMA, HEMA ratio along with the redox initiator CAN. The size of the nanogels decreased with the increase of the CAN amount, however it decreased with the decrease of hydrophilic HEMA ratio. The measured zeta potentials of CsPMH nanogels were in the range of +26.6 and +37.8 mV, which could prevent the aggregation of nanoparticles.

**Figure 4:**
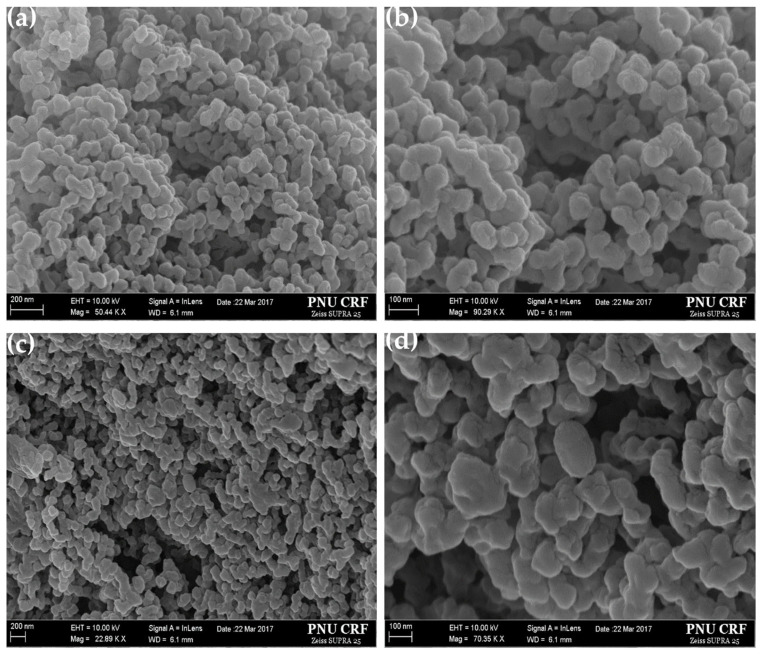
FESEM images of CsPMH nanogels (**a**,**b**) pristine and (**c**,**d**) BPV loaded.

**Figure 5:**
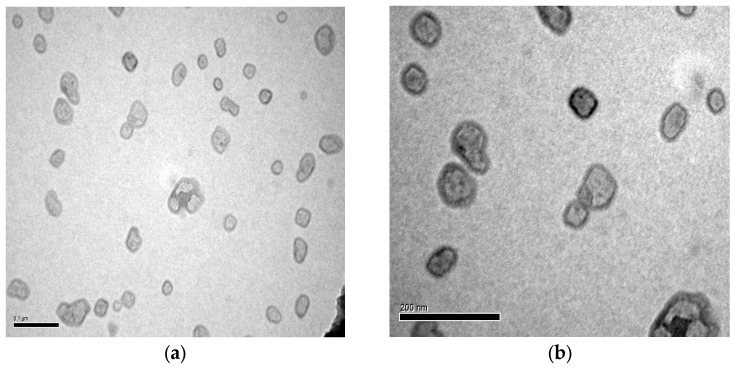
TEM images (**a**,**b**) of pristine CsPMH nanogels.

The electron microscopies, such as SEM and TEM, were utilized to visualize the shapes and morphologies of the nanogels and are displayed in Figure 4 and Figure 5. Figure 4 displays both the pure CsPMH nanogels (Figure 4a,b) and drug loaded CsPMH nanogels (Figure 4c,d). The SEM images showed that the obtained individual particles were aggregated with multi shaped morphology. All particles had almost spherical shapes; however, some deviations between particle sizes determined by SEM and DLS were found, which may be due to the contraction of particles during the drying process of the nanogels. Moreover it should be noted that the particle sizes obtained by the DLS method were the hydrodynamic diameter of the swollen nanogels. When comparing the particle sizes of Figure 4b,d, there was no changes in the morphology of CsPMH nanogels after the BPV drug was loaded. We did notice that there was a small increase in the size of the particles, which could be due to the loading of drug into the free space of the nanogels. Similarly, Figure 5a,b displays TEM images of the pure CsPMH nanogels at different magnifications. As shown in Figure 5, the nanogels were dispersed homogeneously and show some wrinkles, which could be formed during the drying process of the nanogels.

### 2.2. Computational Study

**Figure 6:**
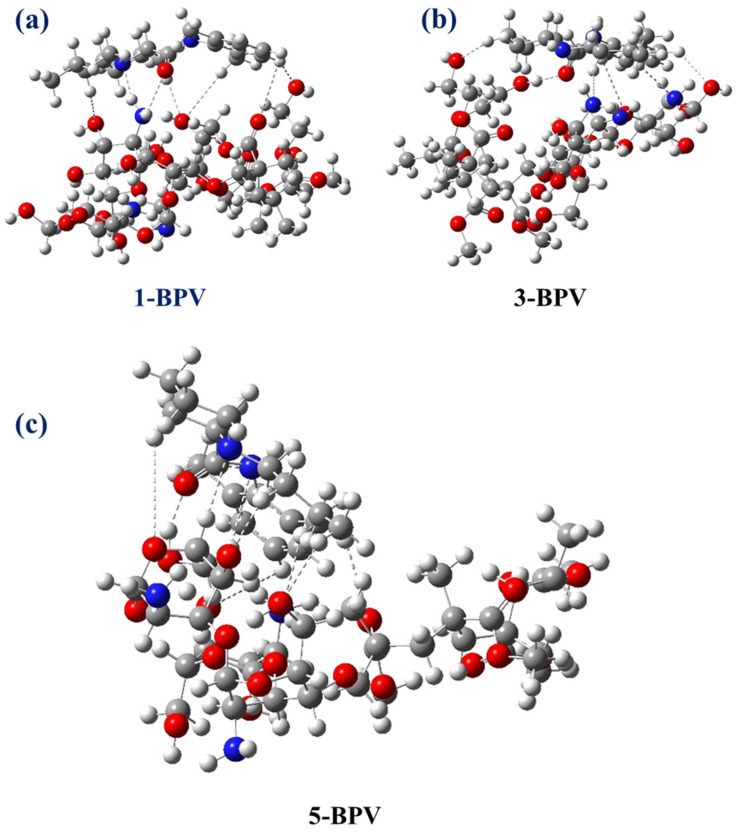
Optimized geometries of 1BPV, 3-BPV, and 5-BPV complexes obtained at M06-2X/6-31G(d) level of theory. Hydrogen bonds are represented by dotted lines. White, gray, blue, and red color spheres represent hydrogen, carbon, nitrogen, and oxygen atoms, respectively.

**Figure 7:**
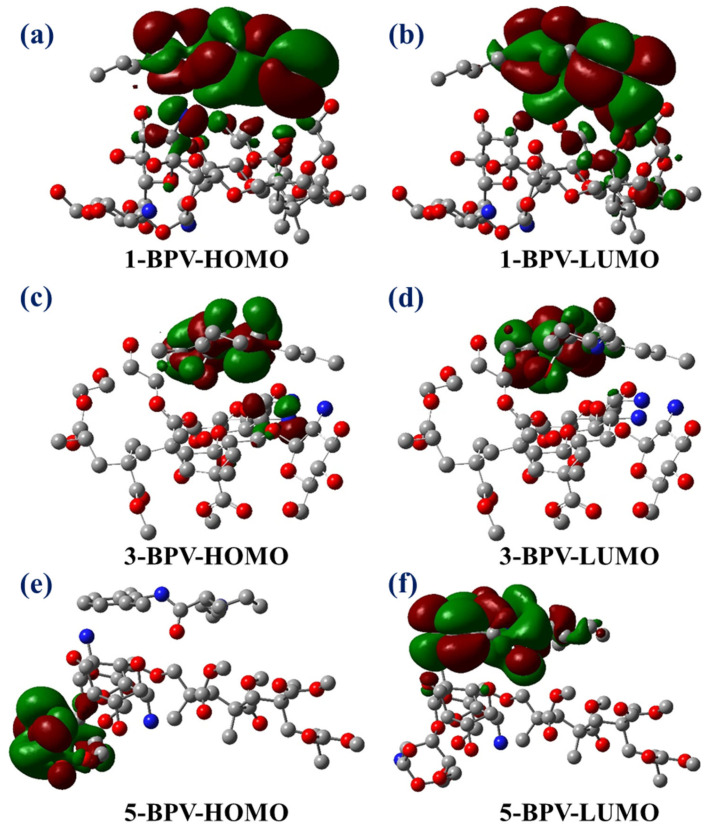
Observed charge transfer phenomenon in frontier molecular orbitals of the complexes obtained at M06-2X/6-31G(d) level of theory. Hydrogen atoms are omitted here for clarity. White, gray, blue, and red color spheres represent hydrogen, carbon, nitrogen, and oxygen atoms, respectively.

Density functional theory (DFT) simulations using the Gaussian 16 program were performed to estimate the binding strength between drug carriers 1, 3, and 5 of Table 4 and BPV drug [42]. The geometries of BPV and three drug carriers were optimized using M062X functional [43] and 6-31G(d) basis set. The selected hybrid meta-GGA-based Minnesota functional (M062X) has been shown to perform well for non-covalent interactions [44]. The complexes formed from the BPV and each of the drug carriers were optimized at the M062X/6-31G(d) level of theory and their local minima were confirmed using vibrational frequency analysis with no imaginary frequency. The complexation energies were corrected for basis set superposition error using counterpoise correction. The complexation energies obtained for the drug carriers 1, 3, and 5 with BPV are −86.92, −71.67, and −97.07 kJ/mole, respectively. The optimized geometries of the complexes and the intermolecular hydrogen bonds are shown in Figure 6. The high complexation energies can be attributed to the intermolecular hydrogen bond interactions formed between BPV and the drug carriers. It is interesting to observe that the number of H-bonds formed in each complex varies: nine for 5-BPV, six for 3-BPV, and seven for 1-BPV. The H-bonds in each complex were formed between different atoms: hydrogen and oxygen, or nitrogen and hydrogen. In the complexes of 5-BPV, 3-BPV, and 1-BPV, there were six, four, six hydrogen bonds of the former molecule, and three, two, one hydrogen bonds of the latter. The charge transfer phenomenon observed in the frontier molecular orbitals, shown in Figure 7, confirms the strong interaction between BPV and drug carrier. The theoretical simulation results confirm that the efficiency of drug carrier 5 was higher than that of the other two drug carriers.

An H-bond is considered to be formed if the distance is less than 4 Å [45]. All the established H-bonds showed shorter distances than 4 Å. In the case of carrier 1, one H-bond was formed between a hydrogen atom on a methoxy group and a nitrogen atom on the BPV, and another H-bond was formed between a hydrogen atom on a hydroxyl group and an oxygen atom on the BPV. In the case of carrier 3, two similar H-bonds were observed between the hydroxyl groups of carrier 3 and the hydrogens of the methyl groups on BPV. The H-bonds with the nitrogens of carrier 3 were observed with the hydrogens of the alkyl chains on BPV. In the case of carrier 5, the hydrogens on the phenyl ring and alkyl chain established two H-bonds with the oxygen and nitrogen of the BPV, respectively. The keto group also has an H-bond interaction with the phenyl hydrogen of the BPV.

### 2.3. Cytotoxicity Test

**Figure 8:**
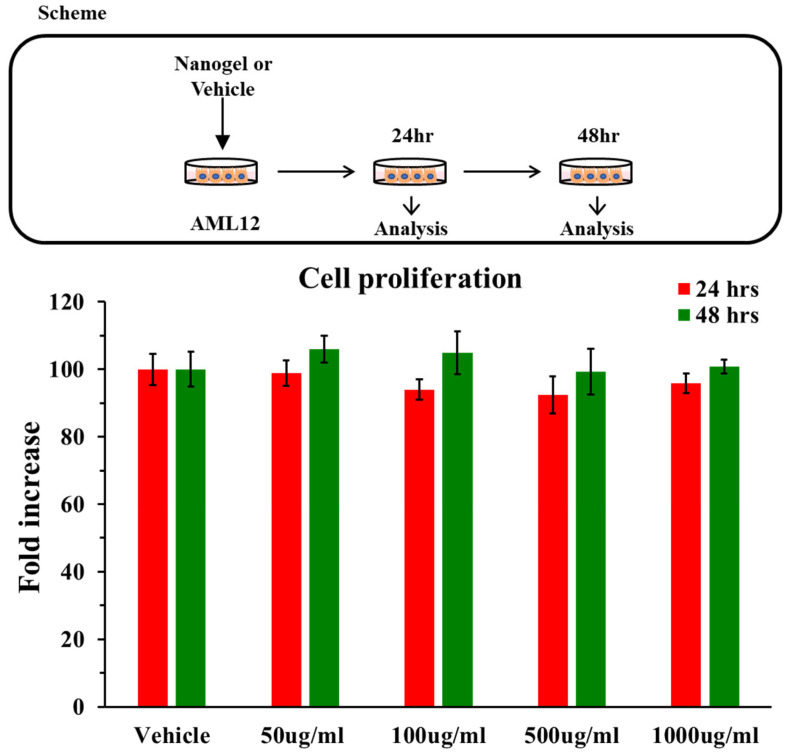
Cytotoxicity assay of AML12 mouse hepatocytes on the CsPMH nanogels (drug carrier-7).

The scheme of analysis and the results of the MTS cytotoxicity test on AML12 mouse hepatocytes are shown in Figure 8. These results indicate that there is no statistically significant difference in the cytotoxicity between drug carrier-7 and the vehicle. It should be noted that the results of both drug carrier-7 and the non-toxic vehicle do not differ in any appreciable way. Hence, the CsPMH nanogels have a natural biocompatibility.

### 2.4. BPV Encapsulation and Release Studies

The functionality of the polymer network, the initial concentration of the polymer, the polymer drug ratio, the types of interactions between drug and polymer, as well as cross-linker, and the swelling ratio are all factors that affect the efficiency of drug encapsulation and release. Among the aforementioned factors, the swelling ratio is the essential one which is closely associated to the drug release. Drug encapsulation was carried out by the batch adsorption method using a freely dissolved solution of BPV, then the resulting BPV loaded CsPMH nanogels were used to evaluate the in vitro BPV release studies after quantification with drug encapsulation and loading efficiency. Table 3 presents the BPV EE% and DLC% efficiencies of different drug carriers of CsPMH nanogels and the values of EE% varied from 43.2 to 91.71%. The study shows that EE% and DLC% varied with the amount of MMA content in nanogels for the drug carriers 1–5. The highest EE% (91.77%) of drug carrier-5 is attributed to the highest complexation energy (−97.07 kJ/mol), which is brought about by nine hydrogen bond interactions between drug carrier-5 and BPV as well as the enhanced hydrophobic interactions between nonpolar parts of the PMMA chains and BPV due to the higher composition of PMMA. Drug carrier-1 has an EE% of 77.86%, which is attributed to the −86.92 kJ/mol of complexation energy through seven hydrogen bonds between drug carrier-1 and BPV. On the other hand, drug carrier-3 has the lowest EE% of 64.88% due to the lowest complexation energy (−71.67 kJ/mol) resulting from six hydrogen bonds. The drug carriers 3, 6, and 7 were fabricated by altering the amount of CAN, a grafting initiator, that could generate the grafted polymer branches from the dp-Cs. Theoretically, the number of grafted polymer branches on dp-Cs should increase with the increase of the CAN amount and produce a dense corona, however, further increase of CAN leads to the decrease of the pore volume between the polymer grafts. Thus, as seen in Table 3, both EE% and DLC% increased then decreased on increase of content of CAN which can produce radicals on the Cs where the graft copolymer could propagate [42,43]. Drug carriers 6 and 7 have lower EE% and DLC%, suggesting that sufficient grafting chains are required for optimal results. In the case of drug carrier 6, the lower values of EE% and DLC% were attributed to the decrease of number by increasing the chain length of grafting chains on the Cs; further, the longer chains could enable more folding, which makes it unfit for the drug loading of nanogels [46,47]. Finally, in the case of drug carrier 7, the larger number of side chains on Cs causes steric hindrance, which prevents both the EE% and DLC%.

**Figure 9:**
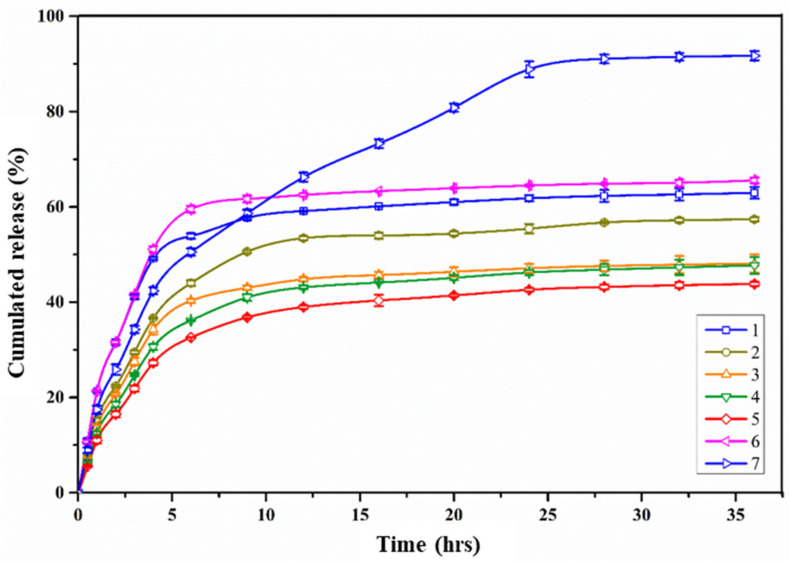
Cumulative release of BPV with different drug carriers of CsPMH nanogels at pH 7.4.

**Figure 10:**
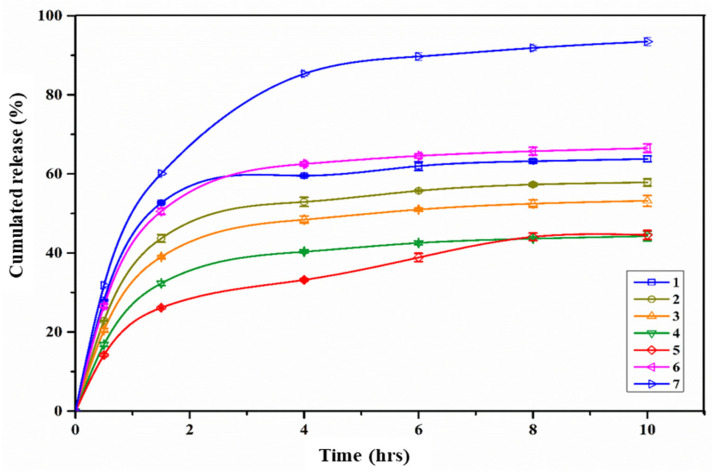
Cumulative release of BPV with different drug carriers of CsPMH nanogels at pH 1.2.

The BPV loaded CsPMH nanogels were subjected to drug release studies at physiological pH condition 7.4 and the release of BPV compared at the lower pH 1.2, and the results are given in Figure 9 and Figure 10, respectively. In the graphs the error bars indicate the mean of the standard error from triplicate drug release data while the data points are mean values. The drug release patterns of all drug carriers are dependent on the feed ratio of monomers and initiator, i.e., the composition and chain lengths of graft polymer on the dp-Cs. At both pH conditions as the amount of MMA amount increased (drug carriers 1–5), the BPV release decreased, which could be due to the increased hydrophobicity of the nanogels. However, the amount of graft chains (drug carrier 3, 6,1 and 7) affects the drug release in a contrasting way to DLC%. Further, the BPV release profile could give the proof of pH dependent behaviors; in acidic medium, a faster release kinetics was observed due to the charged nature of Cs. The drug release with respect to initiator, is unique and shows a decrease of BPV, that means at lowest (20 mg) and highest (80 mg) initiator it leads to higher release rates while with medium (40 mg) initiator it leads to lower rates, which may be attributed to combinations effects like the size of cavities created in the nanogels suitable for trapping more BPV molecules by nanogels and the interaction of the drug with the nanogel matrix. Thus, the drug carrier 7 displayed a controlled release of BPV at both pH conditions, even though it had comparatively lower drug loading capacity. In other words, the max BPV release was found to be significantly higher up to 89% at pH 1.2 in 6 h, while it would reach the same amount of drug release in 24 h at pH 7.4.

These results indicated that, BPV could be released in a more controllable manner at pH 7.4 than 1.2, which could be common for all drug carriers. The stimuli that is responsible for the release of drug at different pH conditions is the ionization of de-acetylated amine groups in the Cs [48]. In neutral condition, the amine groups have neutral changes, allowing the drug molecules to interact with the amines through hydrogen bonding, resulting in slower release rates, whereas in acidic condition, both the amines presented in Cs and drug are cations, leading to repulsive interactions and faster release rates. However, the drug release by the drug carriers 3, 6, and 7 was influenced by the grafting density of poly (MMA-co-HEMA) chains on the dp-Cs, which shows the higher release at 20 mg and 80 mg initiator concentration. This can be explained by the combined effect of polymer folding and steric hindrance for the transport of the BPV from the bulk of the nanogel.

To understand the drug release kinetics and mechanism, different model equations were fitted with the in vitro release data and the results are presented in Table 5 and Table 6 [49]. From Table 5 (based on r^2^ values) it can be shown that the release data were best fitted with the Higuchi equation at pH 7.4 rather than other pHs, meaning that the release of BPV from this system was directly proportional to the square root of time. Further, it indicates that the BPV release from all drug carriers is diffusion controlled. Later, the semi-empirical fitted Korsmeyer–Peppas model was also applied to understand the type of diffusion involved for the BPV release. The “*n*” values in Table 6 describe that the BPV release at pH 1.2 followed the Fickian type diffusion (*n* ≤ 0.45), but anomalous diffusion (0.45 < *n* < 0.89) at pH 7.4, i.e., it could be released by both diffusion and relaxation [49].

## 3. Materials and Methods

### 3.1. Materials

Medium molecular weight Cs (85% deacetylated, Brookfield viscosity 200,000 cps.), MMA (99%), HEMA (96%), ethylene glycol dimethacrylate (EGDMA) (98%) and the model anesthetic drug BPV.HCl (99%) were procured from Sigma-Aldrich (St. Louis, MO, USA). The NaNO_2_, NaOH, methanol, and acetic acid were analytical grade, purchased from Tokyo Chemical Industry (TCI) (Tokyo, Japan) and used without further purification. All the remaining reagents were purchased from Daejung Chemical & Metal (Siheung, Republic of Korea) and used without further purification.

### 3.2. Controlled De-Polymerization of Cs

Cs was chemically depolymerized by a chemical oxidation method using NaNO_2_ solution to obtain the lower molecular weights according to the methods described by Kissel et al. [38] with a minor modification. Briefly, a solution of Cs (2 g of medium-molecular-weight chitosan in 100 mL of acetic acid 6% *v*/*v*) was added to 10 mL of NaNO_2_ solution (70 mg in 100 mL of distilled water) and stirred for 1 h at room temperature. The generated lower-molecular-weight Cs (dp-Cs) was precipitated in aqueous NaOH (4 M) solution with a pH of 9.0 and washed three times with acetone to obtain a white-yellow precipitate. The precipitate was further purified by dialysis (Sigma dialysis tubes MW cutoff 12 kDa) against deionized water with periodic bath changes after being re-dissolved in a minimal volume of acetic acid (0.1 M). The dialysate was freeze-dried from water and kept in storage at 4 °C. The depolymerization of chitosan followed by determination of the weighed average molecular weight of dp-Cs, and its value was 23,800 Daltons.

### 3.3. Nanogels Fabrication

**Scheme 1:**
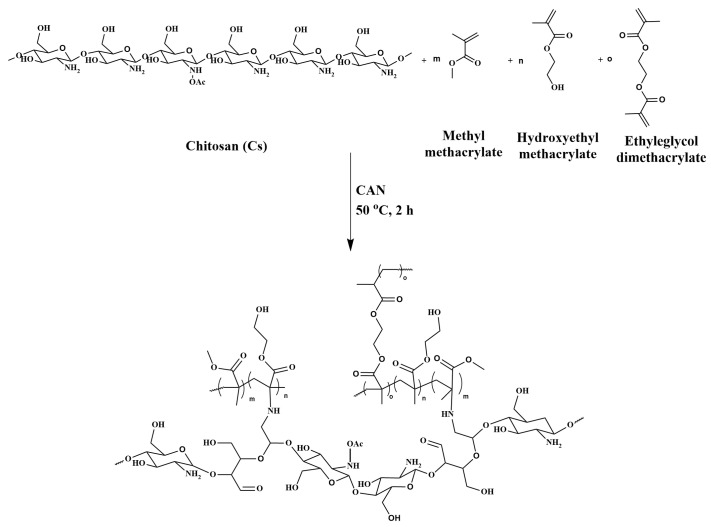
Schematic of CsPMH nanogels from the Cs, MMA, HEMA, EGDMA monomers.

Different drug carriers of Cs/poly (MMA-co-HEMA-cl-EGDMA) (CsPMH) nanogels were fabricated as described below (Scheme 1). According to Table 4, the required amount of dp-Cs was weighed and added to a round-bottomed flask and dissolved in a small amount of 1% acetic acid solution and diluted to 10 mL with distilled water and stirred for about 30 min. Then, the pH of the solution was increased to pH 4 by adding a diluted (0.5 N) sodium hydroxide solution to produce Cs nanoparticles. The solution containing dp-Cs nanoparticles was purged with nitrogen gas for 10 min and heated to the desired temperature on the water bath; ceric ammonium nitrate (CAN) was added to initiate the redox polymerization [50,51]. Then, suitable amounts (as per the Table 4) of MMA, HEMA, and EGDMA were added to support the graft polymerization and crosslinking. After 2 h of reaction the mixture was cooled to room temperature and the nanogels were separated by centrifugation and washed 3–4 times with acetone to remove unreacted monomers then with water. Finally, the obtained nanogels were freeze-dried for further use.

### 3.4. Characterizations

#### 3.4.1. Fourier Transform Infrared (FTIR)

FTIR spectroscopy (4000 to 400 cm^−1^) was used to characterize the functional groups of CsPMH nanogels, using a JASCO spectrophotometer by producing the KBr pellets under a hydraulic pressure of 600 dynes/m^2^. The interpretation of FTIR bands obtained for all samples are tabulated in Table 1.

#### 3.4.2. Thermal Gravimetric Analysis (TGA)

The samples were subjected to TGA using a TGA Q50 (TA Instruments, New Castle, DE, USA) thermo-gravimetric analyzer under nitrogen (40 mL/min) at a scan rate of 10 °C/min, from 30 °C to 800 °C, to study their thermal degradation properties. The interpretation of various steps of weight loss in the thermograms are tabulated in Table 2.

#### 3.4.3. Dynamic Light Scattering (DLS)

DLS techniques were used to measure the particle size and distribution of the CsPMH nanogels. The nanogels were dispersed in double distilled water and the particle size measurements performed at 25 ± 1 °C, on a Malvern Zetasizer nano-S90, (Malvern Panalytical Ltd., Malvern, UK) apparatus equipped with a red laser (λ-633). The triplicate DLS measurements were conducted for each sample and the average of the three measurements displayed. For each sample 10 acquisitions were measured with a 10 s acquisition time. Similarly, zetapotential of appropriately diluted samples (1 mg/mL in water) were measured by using a Malvern Zetasizer nano ZSP instrument (Malvern Panalytical Ltd., Malvern, UK), at 25 °C with detection angle of 90°.

#### 3.4.4. Electron Microscopy

The morphological studies of CsPMH nanogels were performed using SEM and TEM investigations. SEM was carried out using a Carl Zeiss AG-Supra 25 (Jena, Germany) instrument with thin palladium-gold alloy coated CsPMH nanogels, then examined by scanning electron microscopy operating at an acceleration voltage of 15 kV.

TEM was performed at an accelerating voltage of 200 kV using a Hitachi H7600 TEM instrument (Hitachi-High Technologies Corporation, Tokyo, Japan). The samples were made by dropping 10 µL of ultra-sonicated nanogels dispersions on a copper grid, removing the excess moisture with filter paper, and allowing the samples to air dry at room temperature.

#### 3.4.5. Computational Study

The calculations of density functional theory were made using Gaussian 16 software, with a Becke’s three-parameter hybrid functional called B3LYP. The optimized molecular structures of DOX and DCFs (4 repeating units of polymer and CTS rings) and corresponding vibrational assignments of DOX and CTS/PHCH nanogels were investigated with 6-31G(d) basis set and M062X functional.

#### 3.4.6. Cytotoxicity Test

The in vitro cytotoxicity test was measured with a Cell Titer Proliferation Assay (MTS; Promega, Madison, WI, USA) in accordance with the manufacturer’s instructions. In short, pHEPs were plated in 96-well plates at a density of 3 × 10^3^ cells/well and treated with either vehicle (autoclaved triple-distilled water) or suitable concentrations of CsPMH nanogels (drug carrier-7), namely 50, 100, 500, and 1000 μg/mL, for 24 and 48 h. After the treatment, 10 μL of MTS reagent was added to each well, and the plates were incubated at 37 °C in a CO_2_ incubator until the color developed. Finally, the absorbance was measured at 490 nm using a Glomax multidetection system (Promega).

#### 3.4.7. Drug Loading, Delivery, and Model Fitting

BPV loading into the CsPMH nanogels was achieved by using the equilibrium swelling method as per the previous procedure [52]. In detail, the required amount of BPV.HCl was dissolved in water and 2 mL of this solution added to 300 mg of pre-swollen CsPMH nanogels and allowed to absorb the drug molecules. Then the drug loaded nanogels were separated by centrifuge and washed two times with water then dried for further use, while the supernatant solution was used to quantify the remaining BPV after absorption by the nanogels. Based on the optical density obtained by UV-Visible spectrometry, the percentage encapsulation (% EE) and percentage drug loading capacity (%DLC) were calculated using the following equations. After being loaded with free BPV, the CsPMH nanogels were characterized in terms of FTIR, DLS, TGA, drug loading efficiency, and drug release properties. The particles were smaller in size with higher drug entrapment, which could enter the target tissue using the facile EPR effect. Furthermore, the stimuli responsive nature of the nanogels makes it a very effective drug release system.

(1) $$\%\;EE=\frac{Initial\;weight\;of\;BPV-Weight\;of\;BPV\;supernatant}{Initial\;weight\;of\;BPV}\times 100$$

(2) $$\%\;DLC=\frac{Initial\;weight\;of\;BPV-Weight\;of\;BPV\;supernatant}{weight\;of\;CsPMH\;nanoparticles}\times 100$$

BPV loaded CsPMH nanogels (30 mg) were suspended in 5 mL of phosphate buffer solution (PBS) or in 0.1 M HCl in a vial and incubated on a continuous shaker at 37 °C for 3 days. The release medium was filtered with a 0.45 μm PTFE syringe filter to obtain a clear drug solution, which was then quantified using spectrophotometry by measuring the absorbance at 205 nm for predetermined time intervals. Here, the interference of solvent and polymeric nanogels were annulled, using the nanogel equilibrated media as a blank solution. At each time point, a fresh release medium (2 mL) was replaced in the vials with CsPMH nanogels in order to maintain the sink conditions.

Mathematical models are crucial for improving drug delivery system (DDS) design and comprehending the mechanism of drug release [53]. In order to achieve this, we looked at the fitting of various mathematical models of drug release for the initial four hours of experimental drug release data [49,54]. The regression coefficients of the fitting models are shown in Table 4 and Table 5, which indicated that the Higuchi model, which represents the drug release mechanism based on the diffusion-based Fick’s rule employing the square root of time, was the best fit among all the fitting models. This theory states that swelling and dissolving are low in a release environment with optimum sink conditions because the drug particles are substantially smaller than the thickness of the system and the initial drug concentration in the matrix is much greater than the drug solubility. This model, which may be used for medications that are released from various matrices and are both lipid- and water-soluble, is depicted as follows:

(3) $$Q_{t}=\sqrt{D\left(2C-C_{sol}\right)C_{s}t}$$

where *Q_t_* is the amount of drug released from unit area at time *t*, *C* is initial concentration of the drug, *C_sol_* is the solubility of the drug, and *D* is the diffusion constant. As the terms except t are constants, the above equation can be simplified as follows:

(4) $$Q_{t}=\frac{M_{t}}{M_{\infty }}=K_{H}\sqrt{t}$$

where the *M_t_* is and *M*_∞_ are drug released at time *t* and infinite time, and *K_H_* is the Higuchi dissolution constant.

Further, we fitted with other mathematical models of 0 order and first order models as follows:

(5) $$Q_{t}=Q_{0}-k_{0}t\;$$

(6) $$Q_{t}=Q_{e}\left(1-e^{-k_{1}t}\right)$$

Furthermore, we conducted an analysis using the Korsemeyer–Peppas model fitting to gain insight into the mechanism of drug release. This model involves plotting the logarithmic value of cumulative release against the logarithm of time (*logt*) and interpreting the drug release mechanism through the exponential constant “*n*”:

(7) $$\frac{M_{t}}{M_{\infty }}=k_{p}t^{n}$$

(8) $$log\;\frac{M_{t}}{M_{\infty }}={logk}_{p}+nlogt$$

where, *M_t_* and *M*_∞_ are the amounts of BPV released at time *t* and infinite time, and *k_p_* and *n* are the kinetic constant and exponential constant for the drug release, respectively.

## 4. Conclusions

This study aimed to prepare and characterize CsPMH nanogels and investigate their ability to deliver BPV using the pH-responsive nature of the prepared nanogels. Various formulations of CsPMH nanogels were synthesized using the graft copolymerization method of MMA, HEMA, and EGDMA onto depolymerized chitosan. Characterization studies using FTIR and TGA confirmed the molecular-level distribution of BPV within the nanogels. Additionally, DFT calculations indicated that BPV drugs exhibited diverse interactions with different nanogels formulations. The drug percentage loading (DLC%) and percentage encapsulation efficiency (EE%) of CsPMH nanogels depended largely on the amount of MMA used in the nanogels synthesis and the homogeneity of the polymers, which was further confirmed by DFT calculations. Subsequently, in the evaluation of in vitro release behavior of BPV loaded nanogels, all the drug carriers followed the K-P mechanism of drug release. At pH 7.4, all the formulations except drug carrier 7, exhibited comparatively faster release rate and attained a plateau within 6–9 h. However, drug carrier 7 exhibited a steady and sustained release of BPV for approximately 24 h. In conclusion, the CsPMH nanogels have the potential to offer controlled local release of bupivacaine and are well suited for perioperative pain management.

## Figures and Tables

**Table 1:** FTIR absorption bands and their interpretation.

Sample Name	Frequencies (cm^−1^)	Functional Group Assignment
BPV.HCl	3510	–O–H stretch (adsorbed moisture)
	3321, 3249	–N–H stretch (amide)
	2960	–CH_3_ stretch
	2700–2500	–N–H–Cl stretch
	1685	–C=C stretch
	1657	Amide 1 stretch (C=O)
	1560	Amide II bend (N–H)
	1474, 1441	–CH_2_ bend
	1374	–CH_3_ bend
CsPMH nanogels	3518	–O–H stretch
	3434	–N–H stretch
	2952	–CH_3_ stretch
	1728	–C=O stretch (COOR)
	1705, 1640	Amide 1 Stretch (–C=O), Amide II bend (–N–H) of partially de-acetylated acetylated chitosan
	1483, 1445	–CH_2_ bend
	1380	–CH_3_ bend
	1152	–C–O–H stretch
	1073	C–O–C stretch
BPV loaded CsPMH nanogels	3426	–N–H Stretch & –O–H stretch merged
	2947	–CH_3_ stretch
	1728	–C=O stretch (COOR)
	1705, 1638	Amide 1 Stretch (–C=O), Amide II bend (–N–H) of partially de-acetylated acetylated chitosan
	1560–1530 (broad)	Amide II bend (–N–H) of BPV
	1478, 1452	–CH_2_ bend
	1385	–CH_3_ bend
	1148	–C–O–H stretch
	1073	C–O–C stretch

**Table 2:** TGA weight loss steps and their interpretation.

Material	Weight Loss Steps (°C)	Weight Loss (%)	Weight Loss Assignment
CsPMH	30–80	1.28	Water loss from the nanogels
	204–375	72.44	Thermal degradation of polymer side chains and chitosan
	375–519	15.24	Decomposition of pre-resulted polymer network
BPV.HCl	30–115	5.22	Water loss from the nanogels
	150–283	93.96	Thermal decomposition of BPV
BPV loaded CsPMH	118–227	27.56	Thermal decomposition of BPV
	227–400	61.80	Thermal degradation of polymer side chains and chitosan
	400–516	5.80	Decomposition of pre-resulted polymer network

**Table 3:** Drug loading and DLS parameters of different drug carriers of CsPMH nanogels.

CsPMH	Size (nm)	PDI	Zeta Potential (mV)	% EE	% DLC
1	323.8	0.32	+29.8	77.86	18.4
2	290.6	0.47	+27.8	72.67	16.4
3	271.7	0.43	+37.8	64.88	14.3
4	237.7	0.51	+26.6	80.25	19.5
5	236.4	0.47	+29.0	91.77	20.8
6	358.5	0.26	+31.9	43.20	12.4
7	224.4	0.46	+33.4	54.51	14.4

**Table 4:** Feed ratios of CsPMH nanogels.

CsPMH	Cs (mg)	MMA (mg)	HEMA (mg)	EGDMA (mg)	CAN (mg)
1	400	0	400	100	40
2	400	100	300	100	40
3	400	200	200	100	40
4	400	300	100	100	40
5	400	400	0	100	40
6	400	200	200	100	20
7	400	200	200	100	80

**Table 5:** Empirical model fittings for different drug carriers of CsPMH nanogels.

B. No/ Model Name	Higuchi *		Hixson –Crowell *		0 Order *		First Order *	
	r^2^	k_H_	r^2^	k_HC_	r^2^	k_0_	r^2^	k_1_
1	0.9974	28.98	0.9827	0.1574	0.9629	11.82	0.9897	0.0715
2	0.9962	20.985	0.9844	0.8835	0.9729	8.7566	0.9889	0.0477
3	0.9926	20.518	0.9957	0.128	0.9764	8.2211	0.9901	0.044
4	0.9953	18.18	0.9834	0.1125	0.9742	7.3228	0.9873	0.0382
5	0.9823	14.063	0.9835	0.2378	0.9757	6.5338	0.9870	0.0333
6	0.9962	30.339	0.9880	0.1754	0.9706	12.252	0.9809	0.0658
7	0.9956	25.127	0.9932	0.2275	0.9732	10.141	0.9912	0.0578

* Fitting parameters from the release data at pH 7.4.

**Table 6:** Korsmeyer–Peppas model fitting parameters for different drug carriers of CsPMH nanogels.

B. No/ Model Name	Korsmeyer–Peppas pH 7.4			Korsmeyer–Peppas pH 1.2		
	r^2^	n	k_kp_	r^2^	n	k_kp_
1	0.9870	0.7061	19.1293	0.9840	0.2983	41.9759
2	0.9829	0.7273	13.5581	0.9038	0.3209	32.1440
3	0.9901	0.7445	12.3851	0.9144	0.3311	28.8470
4	0.9897	0.7305	11.2642	0.9148	0.3354	23.8232
5	0.9897	0.7402	9.9083	0.9756	0.3891	19.8061
6	0.9873	0.7281	19.0458	0.8984	0.3186	37.4111
7	0.9899	0.7254	15.7290	0.9484	0.3857	45.6142

## Data Availability

Data are contained within the article.
